# Modeling of the *Bacillus subtilis* Bacterial Biofilm Growing on an Agar Substrate

**DOI:** 10.1155/2015/581829

**Published:** 2015-08-17

**Authors:** Xiaoling Wang, Guoqing Wang, Mudong Hao

**Affiliations:** ^1^School of Mechanical Engineering, University of Science and Technology Beijing, Beijing 100083, China; ^2^School of Engineering and Applied Sciences, Harvard University, Cambridge, MA 02138, USA

## Abstract

Bacterial biofilms are organized communities composed of millions of microorganisms that accumulate on almost any kinds of surfaces. In this paper, a biofilm growth model on an agar substrate is developed based on mass conservation principles, Fick's first law, and Monod's kinetic reaction, by considering nutrient diffusion between biofilm and agar substrate. Our results show biofilm growth evolution characteristics such as biofilm thickness, active biomass, and nutrient concentration in the agar substrate. We quantitatively obtain biofilm growth dependence on different parameters. We provide an alternative mathematical method to describe other kinds of biofilm growth such as multiple bacterial species biofilm and also biofilm growth on various complex substrates.

## 1. Introduction

A biofilm is an assemblage of microbial cells that is irreversibly associated with a surface and enclosed in an extracellular matrix which are secreted by bacteria [1]. Biofilm is ubiquitous in natural and industrial environment, and it has both good and bad effects on us. Biofilm can cause problems in energy losses due to increased fluid frictional resistance of the hull surfaces and increased heat transfer resistance in heat exchange equipment, device damage, food contamination, and medical infections; however, biofilms also have a lot of applications such as biofiltration of wastewater and remediation of contaminated soil and groundwater [2–4].

Scientists did a lot of work on biofilm, such as biofilm morphology, structures, and growth process. They proposed models on biofilm to better understand its growth. These models are established for two types of biofilms: one is growing in liquid environment and the other is growing on solid nutritive substrate. The difference between two types is the way of nutrient supply. The biofilm in liquid environment is fed on nutrient from the sides, top, and substrate; the models for them described their heterogeneous structures and growth characteristics [5–8], due to osmotic pressure or extracellular polymeric substances (EPS) [9–11]. The biofilm growing on solid nutritive substrate is exposed to air and only fed through bottom substrate, which contains ions salts and nutrient for biofilm growth, and models for this kind of biofilm described a fractal colony with various morphologies through the diffusion-limited aggregation process [12, 13], studying the role of osmotic stress in biofilm spreading based on the physics of polymer solutions [14].

There are some key factors deciding whole biofilm growth, for example, the yield coefficient, which is an important parameter in biofilm growth and degradation processes [15–17], the maximum specific growth rate, which indicates the microbial inherent growth characteristic [18], nutrient diffusion between biofilm and solid substrate or liquid, which is influenced by extracellular polysaccharides and cellular layers, and the biofilm density, which affects diffusion coefficient and thus has an influence on biofilm growth characteristic [19, 20]. We will consider the above four main parameters in our biofilm growth modeling; for simplicity, we assume diffusion coefficient between biofilm and substrate is a constant.

The existing models for biofilm growth on an agar substrate did not analyze influences of the above main factors on biofilm growth. In our paper, we establish a mathematical biofilm growth model on an agar plate based on mass conservation principle, Fick's first law, and Monod's kinetic reaction. From the numerical analysis, we can obtain dynamic evolution of the biofilm thickness, the volume fraction of active biomass and nutrient concentration in the agar substrate, and parameter effect on biofilm growth. Our model can be used as a tool for describing of other kinds of biofilm growth such as multiple bacterial species biofilm and also biofilm growth on various complex substrates.

## 2. Materials and Methods

*Bacillus subtilis* strain NCIB 3610 is used for all experiments. We grow colonies on 1.5 wt% agar gel with minimal media, MSgg, designed to induce biofilm formation: 5 mM potassium phosphate (pH 7); 100 mM MOPS (pH 7); 2 mM MgCl_2_; 700 *μ*M CaCl_2_; 50 *μ*M MnCl_2_; 50 *μ*M FeCl_2_; 1 *μ*M ZnCl_2_; 2 *μ*M thiamine; 0.5% glycerol; 0.5% glutamate; 50 g/mL tryptophan; 50 g/mL phenylalanine. The agar solution is cooled to 55°C before adding the remaining ingredients. We typically use 100 mm diameter Petri dishes containing 12 mL of media to obtain a 16 mm diameter biofilm after two days. The plates are covered with lids and cooled overnight at room temperature and then spotted within 24 h.

The triple reporter* Bacillus subtilis* strain was obtained from the Kolter lab. This strain was created using phage transduction to fuse a fluorescent gene to the featured promoter into the bacterial chromosome following the established procedure [21]: Red fluorescent protein mKate2 is linked to the hag promoter, which is responsible for flagellin production in motile cells. Blue fluorescent protein cfp is linked to the tapA promoter, which produces the amyloid proteins in matrix-producing cells. Green fluorescent protein citrus is linked to the sspB promoter, which is encoded in late period in sporulating cells. When a featured promoter for a phenotype is expressed, the relevant fluorescent protein is also produced. There is no spectrum overlap of the three fluorophores, except for some minor autofluorescence with CFP filter set. The autofluorescence is because of the pigment production of the colony after 48 h; so most of our experiments last only 48 h.

We transfer the bacteria to the agar surface by spotting with 0.1 *μ*L of bacterial culture at OD_600_ = 1; before inoculating the plates, we remove the lids and allow the surface to dry for 5–10 minutes. We allow the drop to dry for 5–10 additional minutes with the lid off until the meniscus of the initial drop is no longer visible and the bacteria are left in a “coffee ring” around the perimeter. For two days' time-lapse movies, we grow biofilm colonies in a Tupperware container stuffed with wet paper towels and sealed around the microscope using Glad Press'n'Seal plastic wrap to prevent evaporation. The temperature of the microscope is maintained at 32°C using heating elements and fans.

## 3. The Biofilm Growth Model

**Figure 1:**
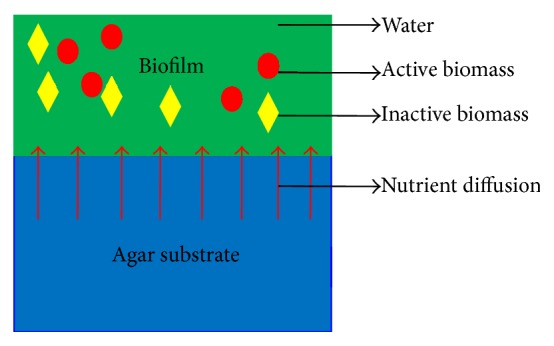
The simplified schematic of biofilm model on the agar substrate. A biofilm is assumed to be made up of active biomass, inactive biomass, and water. The red circle represents active biomass, the yellow rhombus represents inactive biomass, and the green region is water. Nutrient diffuses from agar substrate to biofilm; red arrows indicate the diffusion direction.

We assume the biofilm consists of active biomass, inactive biomass, and water, which are shown in Figure 1; here the biomass is treated as a homogeneous continuum [22]. Inactive biomass is related to endogenous decay and the fraction of the active biomass that is not biodegradable, which also indicates the inert biomass [23, 24]. We consider nutrient diffusion between biofilm and agar substrate, which is shown as red arrows in Figure 1. In our experiment and model, the height of agar substrate is 0.5 cm, and the biofilm area is 1 cm^2^. The volume of the agar substrate (*V*_*a*_) is 0.5 cm^3^.

The unknown dependent variables determined by the model are given in Table 1 and the other variables and the parameters used to develop this model are given in Table 2. Through our paper, the fundamental units of nutrient mass *M*_*n*_, biofilm mass *M*, length *L*, and time *T* are identified. Derivation of biofilm model on agar substrate is shown as in Figure 1.

### 3.1. Mass Balance of Active Biomass

The rate of change of the active biomass depends on the increment rate of active biomass due to cell growth and the inactivation rate of active biomass. The rate of change of the active biomass is given by

(1) ddtρσLtft= Increment rate of active biomass−Inactivation rate of active biomass=VTStK+StYρσLtft−bρσLtft.

The equation can be further reduced to

(2) $$\begin{array}{c} \frac{df\left(t\right)}{dt}=\left(\;\frac{YV_{T}S\left(t\right)}{K+S\left(t\right)}-b\;\right)f\left(\;t\;\right)-\frac{f\left(t\right)}{L\left(t\right)}\frac{dL\left(t\right)}{dt}. \end{array}$$

### 3.2. Mass Balance of Inactive Biomass

The inactive biomass increases as the active biomass becomes inactive. The rate of change of inactive biomass can be given by

(3) ddtσρf¯tLt=Inactivation rate of active biomass=bρσLtft,

which simplifies to

(4) $$\begin{array}{c} \frac{dL\left(t\right)}{dt}=\frac{1}{\bar{f}\left(t\right)}\left(\;bL\left(\;t\;\right)f\left(\;t\;\right)-L\left(\;t\;\right)\frac{d\bar{f}\left(t\right)}{dt}\;\right). \end{array}$$

Adding this to (1) and using the assumption that $f\left(t\right)+\bar{f}\left(t\right)+\varepsilon_{a}=1$ (*ε*_*a*_ is the volume fraction of water in the biofilm) [26], we get the rate of change of the biofilm thickness:

(5) $$\begin{array}{c} \frac{dL\left(t\right)}{dt}=\frac{f\left(t\right)L\left(t\right)Y}{1-\varepsilon_{a}}\frac{V_{T}S\left(t\right)}{K+S\left(t\right)}. \end{array}$$

### 3.3. Mass Balance of Nutrient in Biofilm

The average rate of change of the nutrient in the biofilm depends on the consumption rate of the nutrient and the diffusion rate of the nutrient into the biofilm. A mathematical expression for the rate of change of the nutrient in the biofilm may be given by

(6) ddtStσLt=Diffusion rate of the nutrient−Consumption rate of the nutrient=σDLaSat−St−VTStK+StρσLtft,

which reduces to

(7) dStdt=DLaLtSat−St−ρft+StYft1−εaVTStK+St.

Here, *J* = (*σD*/*L*_*a*_)(*S*_*a*_(*t*) − *S*(*t*)), *J* refers to the nutrient flux through the diffusion layer (*M*_*n*_ *L* ^−2^ *T* ^−1^), according to Fick's first law [22].

### 3.4. Mass Balance of the Nutrient Concentration in the Agar Substrate

The rate of change of the nutrient concentration in the agar substrate is

(8) ddtSatVaDiffusion rate of the nutrient=−σDSat−StLa.

This reduces to

(9) $$\begin{array}{c} \frac{dS_{a}\left(t\right)}{dt}=-\frac{\sigma D\left(S_{a}\left(t\right)-S\left(t\right)\right)}{L_{a}V_{a}}. \end{array}$$

Equations (2), (5), (7), and (9) and initial conditions shown in Table 3 constitute our model. And this model can be simplified to

(10) dftdt=YVTStftK+St1−ft1−εa−bft,dLtdt=ftLtY1−εaVTStK+St,dStdt=DLaLtSat−St−ρft+StYft1−εaVTStK+St,dSatdt=−σDSat−StLaVa.

## 4. Results 

### 4.1. Experimental Results

**Figure 2:**
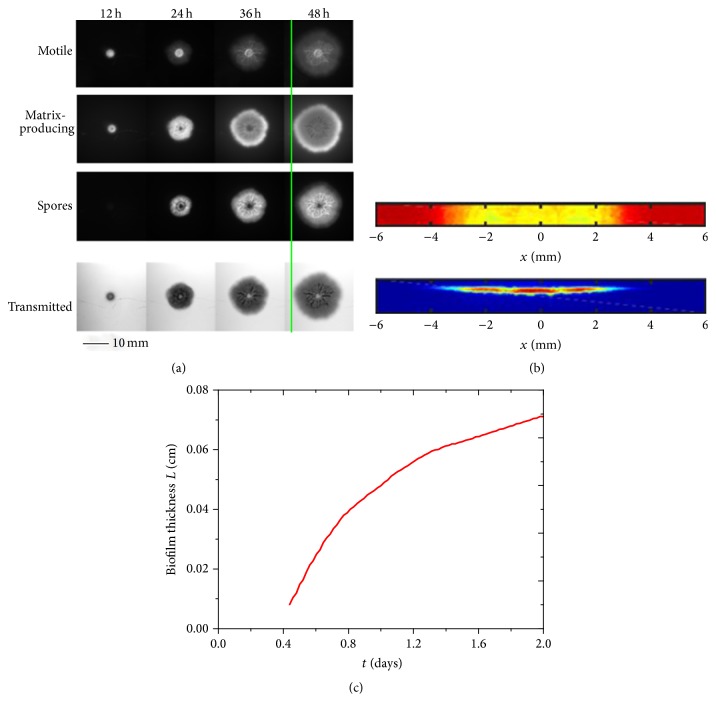
Characterization of growing* Bacillus subtilis* biofilm. (a) Time-lapse images of a growing* Bacillus subtilis* biofilm growing on top of nutritive agar medium at 12-hour intervals. Upon excitation, the motile, matrix-producing, and sporulating phenotypes produce different fluorescent colors and are observed using red, blue, and yellow filters, respectively. The transmission images are shown at the bottom and are used to determine the optical density. Green line serves as guide indicating at 48 hours that the extension of the matrix-producing region is comparable to the motile cells and exceeds that of the sporulating cells.

We make time-lapse movies of the growing biofilm by recording the three fluorescent channels and the transmitted channel. After about 12 h the biofilm becomes visible to the naked eye and continues to grow in a circular fashion such that after 48 h its diameter is 16 mm as shown in Figure 2(a). The images show that three different phenotypes are spatially and temporally organized; details will be described below. To estimate the biofilm thickness from the transmission images, we develop a novel calibration procedure involving cross sections of biofilms. Similar to previous studies [14, 29, 30], we use the Beer-Lambert law to estimate the biofilm thickness *h* from the optical density, OD, through the agar and bacterial colony:

(11) $$\begin{array}{c} OD=\frac{h}{\lambda }, \end{array}$$

where *λ* is the attenuation length. The optical density is defined as

(12) $$\begin{array}{c} OD=-\log_{10}\frac{I}{I_{0}}, \end{array}$$

where *I* is intensity of the transmitted light through the substrate and colony and *I*_0_ is that of the substrate alone, which is transparent agar. To determine *λ*, we compare the transmission (taken from above) with the height obtained from a side view. We reinforce the biofilm for subsequent manipulations by covering it with agar. We cut a thin slab of the biofilm on the agar substrate, whose top view is shown by the transmission image in the top of Figure 2(b). The transmission, *I*/*I*_0_, is determined from the ratio of the transmitted light relative to that of the agar substrate alone and averaged along the narrow, that is, transverse, direction. We next obtain the biofilm's height by flipping the slab onto its side and image its cross section based on the constitutive fluorescent channel as shown in the bottom of Figure 2(b). Finally, we characterize the biofilm's growth based upon the height profiles obtained from the optical density, as shown in Figures 2(c) and 2(b).

Scale bar is 10 mm. (b) Top panel: top view biofilm cross section; here yellow color region is biofilm and red color region is agar substrate. Bottom panel: side view of red constitutive fluorescent labeled biofilm; blue color is agar substrate. (c) Height profile derived from calibration of transmitted images.

### 4.2. Simulation Results

Given the parameters and initial values in Table 3, we numerically solve the main equations (10) by using Matlab package “ODE23s.”

#### 4.2.1. Biofilm Thickness Evolution

**Figure 3:**
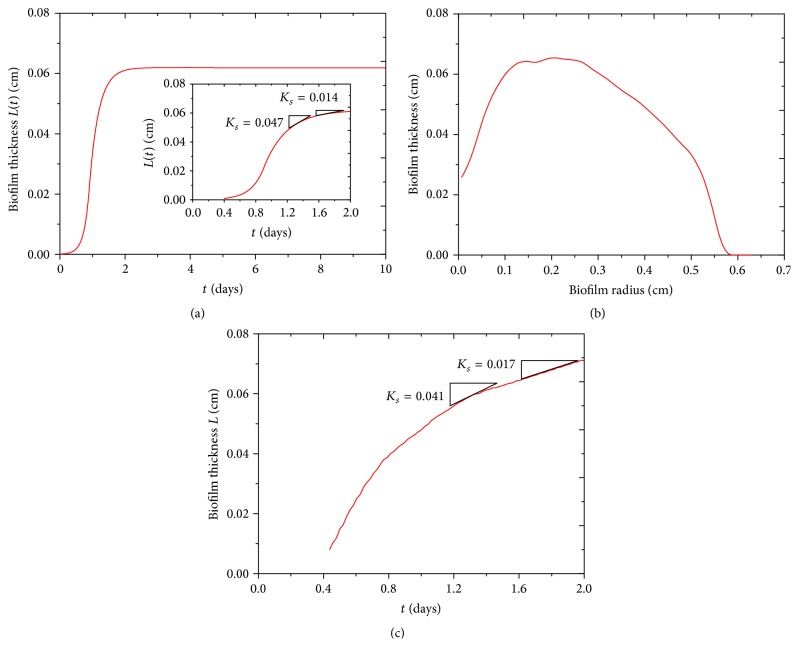
The thickness profile of biofilm. (a) The biofilm thickness in 10 days for biofilm growth model on agar substrate. (b) The biofilm thickness change with its radius in our experiment. (c) The biofilm thickness in 2 days in our experiment. In addition, the rates of biofilm growth in our model and experiment are shown in inset of (a) and (c), which shows a good coincidence.

Biofilm thickness increases from its initial value 0.0001 cm to its steady-state value 0.06 cm, which roughly agrees with our experiment, as shown in Figures 3(a) and 3(c). Biofilm thickness in our experiment firstly increases from its initial 0.025 cm to a maximum and then decreases to zero, as shown in Figure 3(b). By calculating biofilm approximate growth rate through the linear fitting, our results show a good coincidence with our experiment, as shown in inset of Figures 3(a) and 3(c).

In addition, experiments showed that the biofilm resistance to antimicrobial agents was associated with the rate of cell growth in biofilm and biofilm age. The faster the rate of cell growth, the more rapid the rate of inactivation by ciprofloxacin [31]. Anwar et al. [32] found that 10-day-old chemostat-grown* P. aeruginosa* biofilms are significantly more resistant to tobramycin and piperacillin than 2-day-old biofilm. The biofilm stages with different growth rate in our model can be estimated, as shown in Figures 3(a) and 3(c); accordingly, we can test biocides more efficiently.

#### 4.2.2. Volume Fraction of Active Biomass Evolution

**Figure 4:**
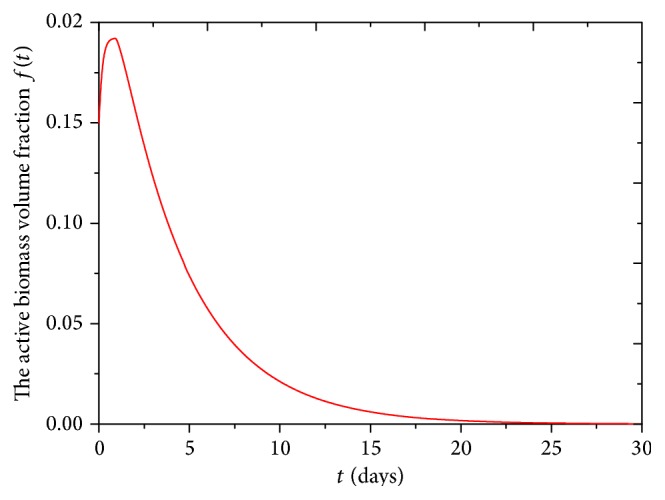
The volume fraction of active biomass in biofilm in 30 days for biofilm growth model on agar substrate.

The volume fraction of active biomass in biofilm increases from initial 0.15 rapidly to maximum 0.19 in about 1 day, which means that the yield rate of active biomass is larger than inactive rate of active biomass in this period. Then volume fraction of active biomass decreases until biofilms are occupied completely by inactive biomass, as shown in Figure 4.

#### 4.2.3. Nutrient Concentration in the Agar Substrate Evolution

**Figure 5:**
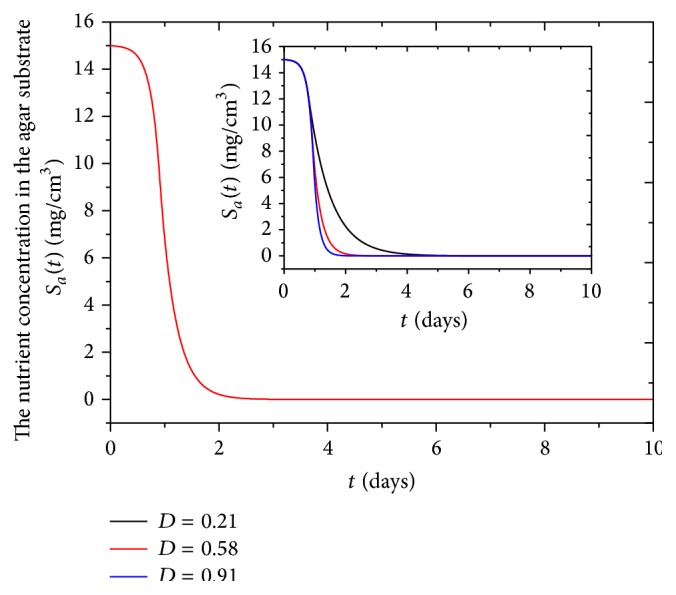
The nutrient concentration in the agar substrate in 10 days for the biofilm growth model. Inset: the nutrient concentration in agar substrate changes with diffusion coefficient *D*  (*L* ^2^ *T* ^−1^).

Nutrient concentration in the agar substrate decreases all the time due to nutrient consumption by biofilm continuous growth and ultimately decreases to zero, as shown in Figure 5. At about 2.5 days, the nutrient concentration in the agar substrate decreases to zero and simultaneously the biofilm thickness increases to a steady-state value due to the nutrient depletion, as shown in Figure 3(a). The rate of nutrient consumption in agar substrate increases with the increase of nutrient diffusion coefficient, as shown in inset of Figure 5.

#### 4.2.4. Effect of Influenced Parameters on Biofilm Growth

**Figure 6:**
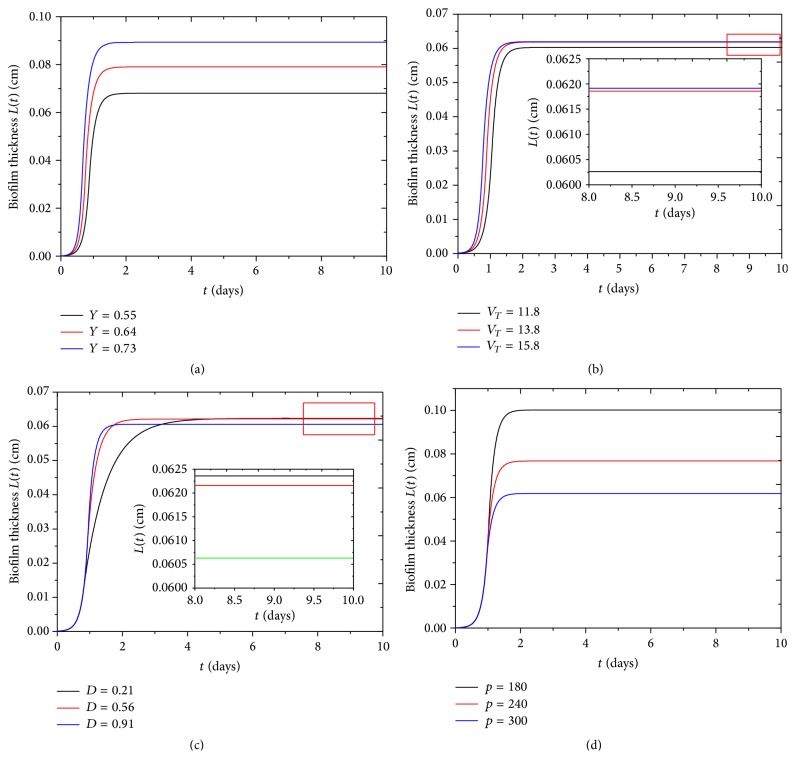
The parameter effect on the biofilm thickness. (a) The yield coefficient *Y*  (*MM*_*n*_^−1^) describes the ratio of the amount of biomass produced to the amount of substrate consumed [25]. (b) The maximum specific growth rate *V*_*T*_  (*M*_*n*_ *M* ^−1^ *T* ^−1^) describes the maximum proliferation ability of microorganisms. (c) The diffusion coefficient *D*  (*L* ^2^ *T* ^−1^) reflects the nutrient diffusion ability into biofilm and (d) the biofilm biomass density *ρ*  (*ML* ^−3^) indicates the biomass in unit volume. Moreover, the biofilm thickness as a function of time for different (b) *V*_*T*_ (*M*_*n*_ *M* ^−1^ *T* ^−1^) and (c) *ρ* (*ML* ^−3^) at a steady state (inset) is shown.

**Figure 7:**
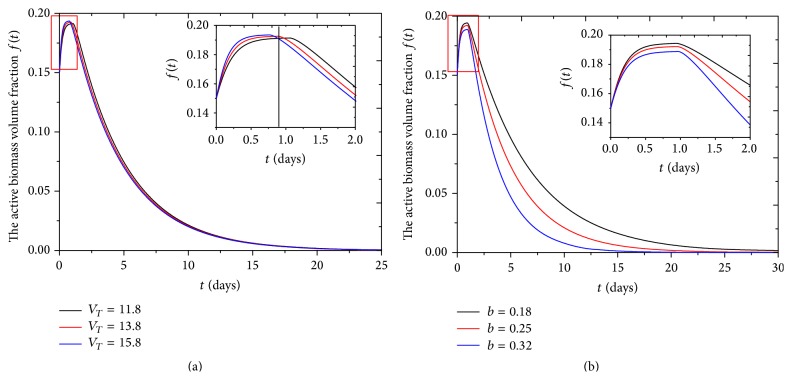
The active biomass volume fraction *f*(*t*) changes with (a) the maximum specific growth rate *V*_*T*_  (*M*_*n*_ *M* ^−1^ *T* ^−1^) in 25 days and (b) inactivation coefficient *b*  (*T* ^−1^) in 30 days; the insets show the change of active biomass volume fraction *f*(*t*) with the two kinds of parameters in 2 days.

We find that when yield coefficient *Y* and the maximum specific growth rate *V*_*T*_ increase, the biofilm thickness increases accordingly, as shown in Figures 6(a) and 6(b), while yield coefficient *Y* has significant effect on biofilm final thickness, as shown in Figure 6(a). It was approved that a larger yield coefficient *Y* would enlarge cell growth rate, and increase of the maximum specific growth rate *V*_*T*_ would increase time-rate-of-change of biomass [33]. The maximum specific growth rate *V*_*T*_ can have positive effect on the maximum value of active biomass volume fraction *f*(*t*) during the first day; at the same time, active biofilm volume fraction *f*(*t*) decreases more rapidly with higher value of *V*_*T*_ after the first day, as shown in inset of Figure 7(a). The reason is that the higher the *V*_*T*_ is, the more the bacteria multiply, which causes more nutrient consumption and competition between bacteria cells. Active biomass volume fraction *f*(*t*) also decreases with increase of rate of active biomass inactivation *b*, as shown in Figure 7(b). Similarly, Kluge et al. found that the inactivation rate of active biomass *b* can cause a negative growth rate of active biomass [34].

The biofilm thickness increases with increase of the diffusion coefficient *D* at first 1.5 days, as shown in Figure 6(c). When the diffusion coefficient *D* increases, the transportation rate of nutrient increases, and biofilm grows rapidly. At the same time, the time needed to nutrient complete depletion decreases, as shown in inset of Figure 5. Gonpot et al. [35] also found that an increase in diffusion coefficient *D* favours growth of the biofilm. We find that when the biofilm reaches the steady state, the thickness is inversely proportional to diffusion coefficient *D*. Because bacteria cannot fully absorb all nutrients from agar substrate when nutrient diffusion coefficient *D* is high, the differences of final biofilm thickness with various diffusion coefficients are slight at 0.002 cm. Biofilm thickness is inversely proportional to biofilm density *ρ*, as shown in Figure 6(d). Liu found that active biofilm thickness decreases significantly with the increased biofilm density *ρ* [36].

## 5. Conclusion and Discussion

We obtain the biofilm thickness and the volume fraction of active biomass in biofilm evolution from our model. The biofilm thickness from our model is comparable with experimental result, which is shown in Figure 3. In fact, the biofilm thickness is inhomogeneous in both azimuthal and radial directions; it is much thicker between center and edge, as shown in Figures 2(b) and 3(b).* Bacillus subtilis* cells can differentiate into multiple phenotypes with different functions during biofilm formation, the main phenotypes including motile, matrix-producing, and sporulating types. We make time-lapse movies of the growing biofilm by recording the three fluorescent channels and the transmitted channel. We observe that the motile cells stay in a circular region about the inoculation spot, as shown in the second row of Figure 2(a). Matrix-producing cells grow radially outwards from the motile region as shown in the third row of Figure 2(a). Sporulating cells occur in a region inside the ring of the matrix-producing cells and outside the region of motile cells, as shown in the last row of Figure 2(a). There are complicated wrinkle patterns distributed in biofilm, which can transport water and nutrient; reasons for wrinkles structure formation are vertical force induced by cell death and lateral force induced from active cells. Wrinkle patterns also depend on mechanical properties of agar substrate and biofilm itself [37–39].

In our model, we obtain the quantitative relationship between some parameters and biofilm evolution; we can control biofilm growth by adjusting these parameters in future experiment. To get biofilm with different thickness and active biomass volume fraction, firstly we can adjust biomass yield coefficient by changing the amount of oxygen in biofilm [40, 41], biomass decay, and different nutrient concentration [2, 42]. Secondly, we can adjust diffusion coefficient and biofilm density [20, 43]. The biofilm density depends on many factors, such as different types of microorganisms [44], physical forces created by hydrodynamic conditions [45], increasing detachment forces related to particle-particle collisions [46, 47], and EPS [48]. Finally, we can adjust the maximum special growth rate of Monod equation by using different microbial species and initial nutrient concentration in agar substrate [49, 50].

In our model, we think nutrient acquisition of biofilm growth mainly depends on nutrient diffusion; the experiment found that the EPS triggering mechanism might bring in nutrient more efficiently than diffusion [29]. We need to consider this point for complete biofilm growth model.

Biofilm growth is complicated and is affected by many factors, including the specific bacteria strain [51, 52], material surface properties, and environmental parameters such as the pH and temperature [1]. To better understand its growth, we need to consider the influence of biofilm channels on biofilm growth, especially the effect of nutrient convection on biofilm thickness and radial expansion.

## Figures and Tables

**Table 1:** Unknown dependent variables and their fundamental units in the biofilm growth model on agar substrate.

Variables	Physical quantity	Units
*L*(*t*)	Biofilm thickness	*L *
*S*_*a*_(*t*)	Nutrient concentration in the agar substrate	*ML* ^−3^
*S*(*t*)	Average nutrient concentration in the biofilm	*ML* ^−3^
*f*(*t*)	Average volume fraction of active biomass in the biofilm	None
$\overset{-}{f(t)}$	Average volume fraction of inactive biomass in the biofilm	None

**Table 2:** Parameters used in the biofilm growth model and their fundamental units.

Parameters	Physical quantity	Units
*σ*	Area of the film-agar interface	*L* ^2^
*D*	Diffusion coefficient for the substrate through	*L* ^2^ *T* ^−1^
	the laminar diffusional sublayer	
*L*_*a*_	Thickness of laminar diffusional sublayer	*L*
*V*_*T*_	Maximum specific growth rate for Monod kinetics	*M*_*n*_ *M* ^−1^ *T* ^−1^
*K*	Monod constant	*M*_*n*_ *L* ^−3^
*ρ*	Biomass density	*ML* ^−3^
*b*	Inactivation coefficient	*T* ^−1^
*Y*	Yield coefficient	*MM*_*n*_^−1^

**Table 3:** Parameter values and initial values for the biofilm growth model simulations on agar substrate.

Term	Value	Units
*K*	0.0001	mg/cm^3^
*V*_*T*_	12.96	1/day [27]
*Y*	0.5	mg/mg
*b*	0.25	1/day
*D*	0.864	cm^2^/day [28]
*σ*	1	cm^2^
*ε*_*a*_	0.8	None
*ρ*	300	mg/cm^3^
*V*_*a*_	0.5	cm^3^
*L*_*a*_	0.3	cm
*S*_*a*_(0)	15	mg/cm^3^ [14]
*L*(0)	0.0001	cm [14]
*S*(0)	0.00004	mg/cm^3^
*f*(0)	0.15	None
